# Proteomic Characterization of *Armillaria mellea* Reveals Oxidative Stress Response Mechanisms and Altered Secondary Metabolism Profiles

**DOI:** 10.3390/microorganisms5030060

**Published:** 2017-09-17

**Authors:** Cassandra Collins, Rachel Hurley, Nada Almutlaqah, Grainne O’Keeffe, Thomas M. Keane, David A. Fitzpatrick, Rebecca A. Owens

**Affiliations:** 1 Department of Biology, Maynooth University, Maynooth, Co. Kildare W23 F2H6, Ireland; mrscindy@gmail.com (C.C.); rachel.hurley.2014@mumail.ie (R.H.); Nada.almutlaqah.2017@mumail.ie (N.A.); grainneokeeffe@gmail.com (G.O.); david.fitzpatrick@mu.ie (D.A.F.); 2 European Bioinformatics Institute, Hinxton, Cambridge CB10 1SD, UK; tk2@ebi.ac.uk

**Keywords:** *Armillaria mellea*, basidiomycete proteomics, oxidative stress, fungal proteomics, methionine synthase, polyamines, secondary metabolism

## Abstract

*Armillaria mellea* is a major plant pathogen. Yet, the strategies the organism uses to infect susceptible species, degrade lignocellulose and other plant material and protect itself against plant defences and its own glycodegradative arsenal are largely unknown. Here, we use a combination of gel and MS-based proteomics to profile *A. mellea* under conditions of oxidative stress and changes in growth matrix. 2-DE and LC-MS/MS were used to investigate the response of *A. mellea* to H_2_O_2_ and menadione/FeCl_3_ exposure, respectively. Several proteins were detected with altered abundance in response to H_2_O_2_, but not menadione/FeCl_3_ (i.e., valosin-containing protein), indicating distinct responses to these different forms of oxidative stress. One protein, cobalamin-independent methionine synthase, demonstrated a common response in both conditions, which may be a marker for a more general stress response mechanism. Further changes to the *A. mellea* proteome were investigated using MS-based proteomics, which identified changes to putative secondary metabolism (SM) enzymes upon growth in agar compared to liquid cultures. Metabolomic analyses revealed distinct profiles, highlighting the effect of growth matrix on SM production. This establishes robust methods by which to utilize comparative proteomics to characterize this important phytopathogen.

## 1. Introduction

Through the Joint Genome Institute (JGI) and MycoCosm, more fungal genomes are becoming available. Currently there are 833 fungal genomes accessible through this resource, including 287 from the Basidiomycota (July 2017). With more genome sequencing projects in the pipeline (1000 Fungal Genomes Project, http://1000.fungalgenomes.org), there are increasing resources for genomic investigations of plant pathogenic fungi. In spite of this, there has been a dearth of investigations utilising proteomic and/or metabolomics tools for basidiomycete characterization. To address this shortfall, proteomic profiling of the plant pathogen *Armillaria mellea* was undertaken to map changes induced by conditions relevant to the ecological niche of this fungus. Namely, the response of *A. mellea* to oxidative stress has thus far remained uncharacterized. Investigation of this condition can contribute to our understanding of the mechanisms utilized to respond to reactive oxygen species (ROS) generated during lignin degradation by this white-rot fungus.

Reactive oxygen species (ROS) are derived from the sequential reduction of molecular oxygen to water and can be generated in organisms by aerobic respiration [1]. ROS are localised in cellular compartments and are scavenged by antioxidants to maintain steady state levels [2], but at high concentrations, they cause wide-ranging cellular damage [3]. They are also utilised as intracellular signalling molecules and in symbiotic and pathogenic interactions [2]. Moreover, in certain basidiomycetes, extracellular lignin degradation is mainly achieved by oxidation reactions catalysed by extracellular oxidases and peroxidases, using hydrogen peroxide (H_2_O_2_) as a co-substrate [4]. Consequently, there are potentially three sources of ROS in *Armillaria mellea* (i) intracellular ROS generated as a result of metabolism; (ii) ROS generated by external stressors, hosts or pathogens; and (iii) ROS secreted by the organism as a means to degrade polymeric and aromatic substrates. 

ROS, particularly hydroxyl radicals, interact non-specifically with all intracellular molecules: proteins, lipids, DNA, RNA and carbohydrates. Many organisms exhibit an oxidative stress response, which initiates a signalling cascade, leading to upregulation of the transcription of antioxidant genes and alterations in cell membrane permeability [5]. ROS are scavenged by small soluble molecules such as polyamines, glutathione, flavonoids and ascorbic acid, which become oxidised by ROS, thus removing the damaging species. Enzymes upregulated under oxidative stress include superoxide dismutase (SOD), which dismutates O_2_^•-^ to hydrogen peroxide, and catalase, which degrades H_2_O_2_ to H_2_O [2]. White-rot fungi, such as *A. mellea*, generate highly reactive ROS that react with lignin aromatic polymers, which are ultimately degraded to CO_2_ and H_2_O [6,7,8]. Lignin-modifying enzymes, laccases, heme-containing peroxidases, multicopper oxidases and metal-containing oxidoreductases are involved in a network of processes generating ROS to breakdown a wide range of aromatic compounds by oxidative processes.

The two stressors are also alternately dissipated, whereby H_2_O_2_ generates hydroxyl radicals, while menadione generates a range of ROS by the Haber-–Weiss reaction. Menadione, in a redox cycle, generates superoxide ions, which can inhibit iron-sulphur protein activities and produce hydroxyl radicals [9,10,11]. Oxidative stress induced by H_2_O_2_ in *A. nidulans* increased levels of intracellular O_2_^2−^ and O_2_^•-^, whereas menadione-induced stress increased O_2_^2−^ and O_2_^•-^ levels, but also promoted intracellular accumulation of O_2_^2−^ [9]. Consequently, the aims of the work presented here were to carry out the first differential proteomic analyses, by 2-DE, of *A. mellea* by subjecting the organism to two distinct oxidative stress conditions, namely H_2_O_2_ and menadione/Fe^3+^, respectively, and to identify differentially abundant proteins in order to yield information on how *A. mellea* responds to oxidative stress.

In addition to oxidative stress response, further investigation was carried out to characterize the altered proteome of *A. mellea* grown in different culture matrices. Much of the comparative proteomics carried out on filamentous fungi has been carried out on extracts from mycelia grown in static or shaking liquid cultures, or secreted proteins harvested from culture broth [12,13,14,15,16,17]. Imanaka et al. (2010) compared the effect of cultivation method on *Aspergillus oryzae* and noted differences in protease and α-amylase activity, detected following growth in liquid compared to agar culture [18]. Analysis of protein levels showed that trends in enzyme activity were found to correlate with the abundance of the associated enzyme. In contrast, transcriptomic analyses of each of these conditions demonstrated that gene expression did not always correlate with the levels of enzyme detected, thus highlighting the importance of a proteomic approach. Methods for extracting proteins from agar cultures of filamentous fungi have been developed [18,19], but the use of these in differential MS-based analysis has been limited. Key to success in comparative proteomic analyses is a high level of reproducibility, and this is especially important in label-free MS-based proteomics, due to the absence of sample multiplexing. Comparative MS-based analysis was carried out to track changes to the proteome of *A. mellea* grown in liquid or agar cultures, while also validating the reproducibility of protein extraction from agar cultures. Several putative secondary metabolism proteins were detected with altered abundance in agar cultures compared to stationary liquid cultures. These changes were reflected in distinct metabolite profiles from either condition, as determined by RP-HPLC with independent UV and MS/MS analyses. Together, these studies highlight the relevance of proteomic and metabolomics tools in the characterization of plant pathogens in response to environmental changes (stress/growth matrix).

## 2. Materials and Methods

### 2.1. Culture Conditions

*A. mellea* cultures were maintained on malt extract agar (MEA) at 25 °C. To prepare liquid cultures, 3-week agar cultures of *A. mellea* (25 mL) were broken up into 3–5 mm^3^ pieces, and 10 mL of sterile water were added. Agar pieces were vortexed, and 1-mL aliquots of the homogenate were used to inoculate liquid media. *A. mellea* was grown for 28 days in potato dextrose broth (PDB) at 25 °C and was subjected to two distinct oxidative stress conditions: 1 mM H_2_O_2_ or 0.5 mM menadione/100 µM FeCl_3_ for 3 h, after 28 days. Relevant controls were included for each condition. Protein was extracted from two biological replicates per condition for gel-based analyses. For MS-based proteomics, liquid (potato dextrose broth (PDB)) and agar (Potato dextrose agar (PDA)) cultures of *A. mellea* (*n* = 3 biological replicates) were prepared and grown for 3 weeks at 25 °C. Liquid cultures were harvested by separating culture supernatants from mycelia using Miracloth. Mycelia were washed several times with distilled water; excess liquid was removed by blotting between filter paper; and mycelia were snap-frozen in liquid N_2_ [19]. 

### 2.2. Protein Extraction and 2-DE 

Protein extraction was carried out at 4 °C, whereby *A. mellea* mycelia from biological replicates were processed individually as previously described [19]. Briefly, mycelia from independent cultures were ground in liquid N_2_ and 1 g resuspended in 6 mL 10% (*w*/*v*) trichloroacetic acid (TCA) (4 °C) and sonicated six times (Bandelin Sonopuls HD2200 sonicator, Cycle 6, Berlin, Germany, 10 s, Power 10%). Samples were then centrifuged at 20,200× *g* for 10 min (4 °C), followed by washing in ice cold acetone, twice. Pellets were finally resuspended in isoelectric focusing (IEF) buffer (10 mM Tris, 8 M urea, 2 M thiourea, 4% (*w*/*v*) CHAPS, 1% (*v*/*v*) Triton X-100, 65 mM dithiothreitol (DTT) and 0.8% (*w*/*v*) IPG buffer), centrifuged at 14,000× *g* for 5 min (4 °C), and supernatants used for further analysis. Proteins were quantified using the Bradford assay, and 300 µg/strip were used for IEF using 13-cm strips (pH range 3–10; IPGphorII; GE Healthcare, Little Chalfont, U.K.), followed by SDS-PAGE separation on PROTEAN Plus Dodeca Cell (Bio-Rad, Hercules, CA, USA) and Colloidal CoomassieR Blue staining. After destaining, gels were recorded as digital images using a CCD scanner, and image quality was inspected, aligned and replicates grouped using SameSpots™ software (v 4.5.5101.46512). Spots were selected for LC MS/MS analysis based on a statistically-significant difference of *p* < 0.05 (ANOVA) and a fold change ≥1.5 from analysis, as well as visual inspection. In-gel digestions (2-DE spots) were carried out as described previously [19,20] prior to LC-MS/MS analysis. 

### 2.3. Protein Extraction for In-Solution Digestion

Frozen mycelia from PDB cultures were ground in liquid N_2_ and protein extracted as described previously [21] with some alterations. Briefly, 1 g of crushed mycelia was resuspended in 6 mL of lysis buffer (25 mM Tris-HCl, 6 M GdnHCl, 10 mM DTT pH 8.6) and sonicated. Protein extraction from PDA agar plates was performed as described previously [19]. All lysates were clarified twice by centrifugation, and resulting supernatants were brought to 15% TCA. Precipitated protein was pelleted by centrifugation after 30 min, and pellets were washed twice with ice-cold acetone. Pellets were finally resuspended in UT buffer (6 M urea, 2 M thiourea, 0.1 M Tris-HCl pH8). Protein concentration was determined using a Bradford protein assay, and all samples were adjusted to 1 mg/mL in advance of digestion. Trypsin digestion of protein samples was carried out as described previously [22], and sample clean-up was performed using Millipore C18 Ziptips^®^ (Billerica, MA, USA), as per the manufacturer’s guidelines.

### 2.4. LC-MS/MS Identification of A. mellea Proteins

An Agilent 6340 (Santa Clara, CA, USA) ion trap mass spectrometer running ChemStation 0.01.03-SR2 (204) and having a ProtiID-Chip150 C18 150-mm (G4240-62006) chip was used for peptide separation from in-gel protein digests: Solvent A: 0.1% (*v*/*v*) formic acid and Solvent B: 0.1% (*v*/*v*) formic acid in 90% (*v*/*v*) acetonitrile. For protein identification, a linear gradient of 5–70% Solvent B over 7 min (flow rate: 0.6 μL/min) was deployed, followed by washing and re-equilibration steps. LC-MS/MS spectra were analysed using Spectrum Mill software (Rev A.03.03.084 SR4; Agilent Technologies) to identify proteins against the translated *A. mellea* cDNA database [19] (http://genome.jgi.doe.gov/Armme1_1/Armme1_1.home.html). MS/MS searches were carried out with the following settings: enzymatic trypsin digestion, carbamidomethylation as fixed modification and two variable modifications were set: deamination and oxidation of methionine. The minimum scored peak intensity was set to 70; with precursor mass tolerance ±2.5 Da and product mass tolerance ±0.7. The maximum ambiguous charge was set to 3 with a sequence tag length >3 and minimum detected peaks set to 4. Reversed database scores were calculated. For in-solution digests from PDA and PDB cultures, a Dionex 3000 RSLCnano (Sunnyvale, CA, USA) coupled to a Thermo Q-Exactive (Waltham, MA, USA) mass spectrometer was used for peptide separation and identification, respectively. Complex peptide mixtures were separated on a 2-h gradient (3–40% Solvent B) on a 50 cm × 75 µm Easy-Spray PepMap™ column at 0.3 µL/min. Generated spectra were analysed using MaxQuant (v 1.5.3.30) for protein identification, and the label-free quantitation (LFQ) algorithm [23] was used for comparative analysis between liquid and agar extracts using the *A. mellea* protein database obtained from http://genome.jgi.doe.gov/Armme1_1/Armme1_1.home.html [19]. Perseus (v 1.5.4.0) was used for data organization and statistical analyses.

### 2.5. A. mellea Metabolomics

Metabolites were extracted from culture supernatants from PDB liquid cultures or from agar plugs from PDA solid-phase cultures. For liquid cultures, culture supernatant was separated from mycelia using Miracloth and lyophilized to concentrate. Dried samples were resuspended in minimal volume of PBS and passed through 3-kDa cut-off centrifugal filters to isolate low molecular weight molecules. For extraction of small molecules from solid-phase cultures a method was adapted from [24]. Agar plugs (diameter: 8 mm) were taken from various positions in the agar culture and extracted with ethyl acetate for 30 min in a sonication bath. The organic extract was then separated and dried to completion. Prior to LC-MS/MS, all samples were resuspended in methanol and diluted to 10% (*v*/*v*) methanol, 0.1% (*v*/*v*) formic acid. Small molecule samples were analysed by LC-MS/MS using the Agilent 6340 ion trap mass spectrometer as described above. Manual analyses of spectra were carried out to characterize the molecules detected.

### 2.6. Bioinformatic Tools 

*A. mellea* protein coding genes [19] were obtained from http://genome.jgi.doe.gov/Armme1_1/Armme1_1.home.html and saved, with Am1–Am20349 nomenclature, with the sequence alignment editor BioEdit [25]. Annotation and functional analysis was performed using the online software tool Blast2GO (B2G) [26]. Using B2G, a blastp search of the National Centre for Biotechnology Information non-redundant database (NCBInr) was run, with the number of hits set to ten and files saved in .xml format; a GO mapping step followed after which annotation and annotation augmentation were implemented; InterProScan (IPS) and “InterProScan GO’s merge to annotation” was performed; enzyme code and KEGG mapping steps were run, after the completion of which, GO slim was executed and combined outputs generated.

## 3. Results

### 3.1. 2-DE Interpretation

**Figure 1:**
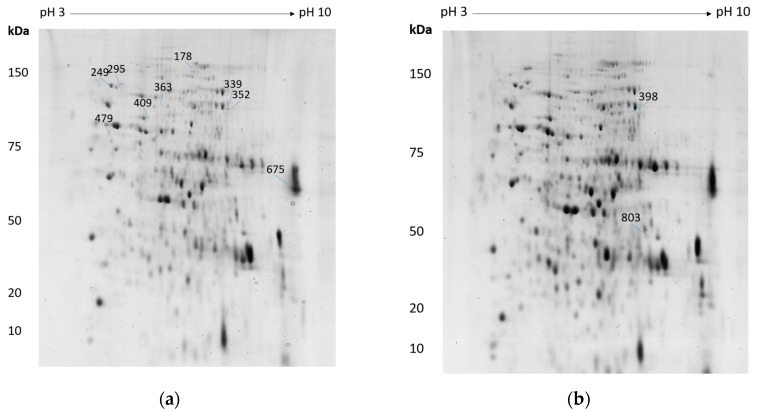
Reference gel images of 2-DE separation of *A. mellea* mycelial protein extracts following (**a**) H_2_O_2_ or (**b**) menadione/FeCl_3_-induced oxidative stress for 3 h. The image shows protein spot numbers from which a single differentially-regulated protein was identified.

The 2-DE reference gel of *A. mellea* mycelial protein extract following H_2_O_2_-induced stress is shown in Figure 1a, with the differentially-regulated, unambiguously-identified spots outlined and numbered. There were 1142 spots identified on the gels, and twenty-six protein spots were found to be differentially regulated in the H_2_O_2_ dataset with a 1.5–2.6-fold change. LC-MS/MS analysis yielded reliable identification from nine spots, correlating to eight unique proteins (Table 1). Cobalamin-independent methionine synthase (Am17277) was identified independently from two spots that were observed to increase in abundance, indicating that two isoforms of this protein were affected by H_2_O_2_ treatment. The 2-DE reference gel of extracted mycelial proteins of *A. mellea* following menadione/FeCl_3_-induced stress is shown in Figure 1b, with differentially-abundant and unambiguously-identified spots outlined and numbered. In the menadione/FeCl_3_ dataset of 1122 proteins that were visualised, nine protein spots exhibited differential abundance whereby the fold change ranged from 1.5–3.5. Protein identifications were only confirmed if a single protein was reliably and unambiguously identified from a spot, and so, only two protein identifications could be assigned following LC-MS/MS analysis (Table 1). Notably, methionine synthase (Am17277) was independently identified with significantly increased abundance in response to both H_2_O_2_ (↑ 2.7, 1.8) and menadione/FeCl_3_-induced oxidative stress (↑ 2.6).

### 3.2. Effect of Culture Matrix on Protein Expression in A. mellea

**Figure 2:**
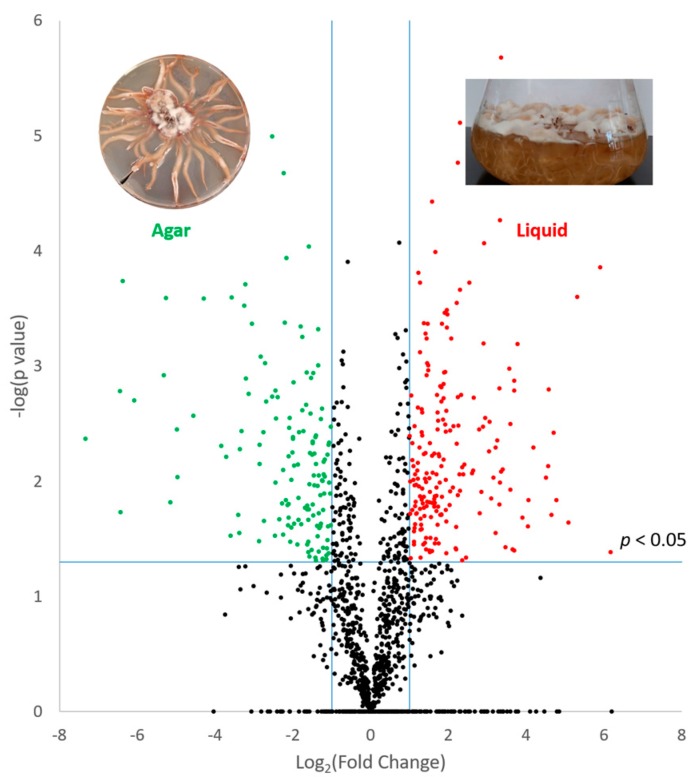
Volcano plot displaying quantitative data from the comparison between proteins detected in liquid and agar cultures of *A. mellea*. Proteins significantly increased in abundance (*p* < 0.05) and with a fold change greater than two are depicted in colour. Red circles indicate proteins increased in agar extracts, and green circles indicate proteins with a higher abundance in extracts from liquid cultures.

Following analysis using MaxQuant software, a combined total of 1984 unique proteins was identified from cultures grown in liquid or agar cultures. This included 1787 proteins detected in extracts from liquid cultures and 1547 proteins detected in extracts from agar cultures. Pearson correlation values within groups of replicates were >0.93, indicating good reproducibility between biological replicates. Qualitative analysis revealed proteins that were uniquely detected in liquid cultures (*n* = 437) or agar extracts (*n* = 197) (Table S1). Label-free quantitative analysis further revealed proteins with significantly higher abundance in either liquid (*n* = 203) or agar (*n* = 144) cultures (*p* < 0.05; fold change ≥2) (Figure 2) (Table S1). Qualitative and quantitative data were combined to determine proteins that were elevated in either growth condition, and bioinformatic analyses were performed on these datasets. Within the subset of proteins that were altered in abundance between the two growth phases, a number of proteins related to secondary metabolism (SM) were identified. The SM cluster resource available through MycoCosm at JGI has putatively assigned 30 secondary metabolite clusters detected in the *A. mellea* genome (http://genome.jgi.doe.gov/Armme1_1/Armme1_1.home.html) [27]. Genes encoding proteins involved in the production of secondary metabolites are clustered in a contiguous fashion in fungi. These clusters consist of genes for a backbone enzyme, such as a polyketide synthase (PKS), as well as accessory proteins involved in further processing or transport of the metabolite. Proteins (*n* = 18) were identified in this study that correspond to these putative secondary metabolite clusters (Table 2). Of these, six proteins were detected exclusively in the agar extracts with a further protein, fatty acid synthase (Am6587), detected with significantly elevated abundance. From the proteins with increased abundance in the liquid cultures, two putative secondary metabolite cluster members were identified, namely an NAD(P)-binding protein (Am14843) and a protein with an RNA-binding domain (Am19046). Additional secondary metabolism-associated proteins were also detected, which showed no significant change in abundance between the conditions (Table 2). 

### 3.3. Metabolite Profiling of A. mellea in Different Culture Conditions

**Figure 3:**
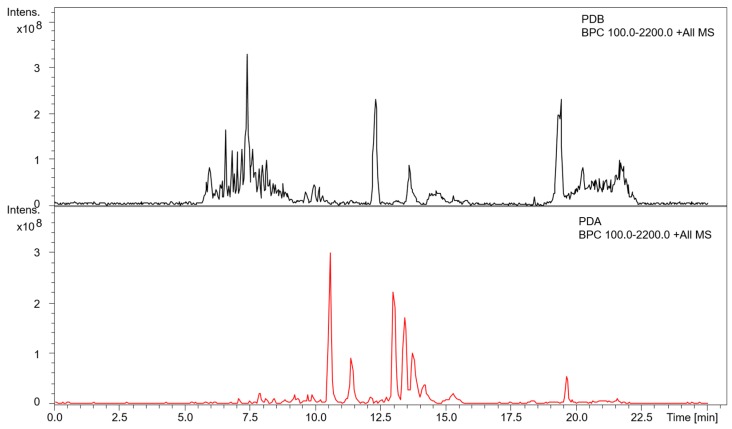
Base peak chromatographs (BPC) of extracts from potato dextrose broth (PDB) liquid culture supernatants and potato dextrose agar (PDA) culture extracts. Intens.: Relative intensity of ions.

Metabolite extracts from liquid and agar cultures of *A. mellea* were profiled by RP-HPLC with a photodiode array detector, in addition to LC-MS/MS analysis for mass determination. Distinct RP-HPLC profiles were noted between extracts from liquid culture supernatants and agar cultures (Supplementary Figure S1). These differences are in line with the altered abundance of secondary metabolism-related proteins observed between the different growth conditions and highlight the influence of growth matrix on metabolite production. Additional characterization of small molecule extracts was performed using LC-MS/MS analysis (Figure 3). Consistent with the RP-HPLC profiles, several additional molecules were detected in the PDA extracts compared to the extracts from PDB liquid culture supernatants. Many of these molecules generated similar signature fragment ions (*m*/*z* 264.8, 246.8), indicating that their structures may be related, possibly differing by modifications on a common core structure (Supplementary Figure S2). An additional molecule (*m*/*z* 404) was detected in both growth conditions, indicating that the production of this molecule was not influenced by the cultivation method.

## 4. Discussion

Comparative proteomic analyses are a critical addition to the toolbox for characterizing plant-colonising fungi, and therefore, the development of robust and reproducible methods for performing these studies is vital. Utilising both a gel-based and an MS-based approach, we have characterized the important phytopathogen *A. mellea* with respect to oxidative stress response and effects associated with the cultivation method, respectively. 

Profiling the proteome of *A. mellea* upon exposure to oxidative stress can illuminate mechanisms within this organism relevant to lignin degradation, in addition to the response to exogenous stress from host/pathogen interactions. Cobalamin-independent methionine synthase (Am17277; Table 1) exhibited increased abundance under both oxidative stress conditions tested. Under H_2_O_2_-induced oxidative stress, two isoforms showed higher abundance (2.7-fold and 1.8-fold) (Table 1), while under menadione/FeCl_3_-induced oxidative stress, there was a 2.6-fold elevation in abundance. Cobalamin-independent methionine synthase catalyses the formation of l-methionine from l-homocysteine and so may represent a vehicle for *A. mellea* to reduce toxic levels of homocysteine. Methionine is also a key substrate for the production of *S*-adenosylmethionine (SAM), the universal cellular methyl donor, which may be required for many reactions in the oxidative stress response. Methionine also protects proteins against oxidative stress [31,32] and was shown to be essential in *C. albicans*, as mutants lacking the methionine synthase (MET6) gene were non-viable even when supplemented with exogenous methionine [33]. Methionine residues are readily oxidised by many ROS and scavenge ROS to form methionine sulfoxide. This, in turn, can be reduced by methionine reductase, found in the majority of cells, to form methionine [31]. Clearly, the involvement of Am17277 in methionine biosynthesis, along with simultaneous homocysteine utilisation, provides a clear rationale for the observed increased expression under both oxidative stress conditions. Methionine, via SAM, is required for chromatin methylation, thereby regulating gene expression, and, as noted above, is required for SAM biosynthesis, which is essential for polyamine synthesis via the action of spermidine/spermine synthase (Am14050; Table 1). This observation strongly suggests an interplay between methionine biosynthesis and polyamine production in *A. mellea*, to produce ROS-scavenging metabolites as a protective mechanism against cellular damage. Cobalamin-independent methionine synthase has also been detected with increased abundance in the white-rot fungus *Phlebia radiata* following growth on spruce wood, which may point to a role in lignocellulose degradation mechanisms [34]. Notably, the cobalamin independent methionine synthase was also detected with increased abundance in yeast following H_2_O_2_ treatment [35]. *Escherichia coli* methionine synthase MetE undergoes inactivation during oxidative stress, resulting in methionine auxotrophy [36,37]. Loss of activity was due to irreversible oxidation of MetE in the presence of H_2_O_2_ or diamide [36,38]. Elevation in levels of Am17277 in *A. mellea*, in response to both forms of oxidative stress, could therefore be a compensation mechanism to restore methionine biosynthesis. 

Am14050, a putative saccharopine dehydrogenase, exhibited 1.7-fold increased abundance under H_2_O_2_-induced stress (Table 1). BLAST2GO analysis identified this protein as having homology to a spermine/spermidine synthase: saccharopine dehydrogenase/homospermidine synthase. Spermine/spermidine synthase is implicated in many metabolic pathways including methionine salvage and polyamine metabolism [39]. Notably, polyamines play a non-enzymatic role in scavenging ROS [2]. Spermine is a polyamine contained in all eukaryotic cells, and some prokaryotes, and is crucial for survival [40]. Spermidine is a mediator of cell proliferation and stabilises nucleic acids [41]. Spermine/spermidine were shown to protect murine cells against H_2_O_2_ oxidative stress by scavenging ROS [39]. A saccharopine dehydrogenase/spermidine synthase signature is specific to basidiomycetes and is a chimera of two genes without an intermediate stop codon or second initiation codon. This gene is multifunctional, with disparate roles in lysine and spermidine biosynthesis, and homologs have been identified in many basidiomycetes [42]. In *Ustilago maydis*, a regulatory role for this gene in polyamine homeostasis by an unknown mechanism has been proposed [43]. Mutant *Ustilago maydis* with deletion of the chimeric gene was more susceptible to stress and less virulent, and the morphology of cells grown in acidic conditions, normally filamentous, was yeast-like and could not be restored by subsequent culture at pH 3–7 [43]. Thus, in *A. mellea*, increased abundance of Am14050 in response to stress may also implicate it in virulence; however, this link would require additional investigation to confirm. Both spermine and spermidine prevent DNA damage and apoptosis, but spermine has been shown to be the more active scavenger of peroxyl radicals even at low concentrations [39,44,45,46]. Exposure to exogenous spermidine was responsible for a wide range of gene upregulation, and exposure of a polyamine-deficient mutant of *Saccharomyces cerevisiae* to spermidine resulted in increased methionine biosynthesis [47]. Conversely, a study in *Aspergillus fumigatus* challenged with H_2_O_2_ found increased abundance of spermine synthase, but downregulation in methionine synthase [48]. As both methionine synthase and spermine/spermidine synthase showed increased abundance in *A. mellea* under H_2_O_2_-induced oxidative stress, this may indicate upregulation of methionine biosynthesis by increased spermidine in *A. mellea*. Alternatively, increased methionine biosynthesis may be required to produce SAM, and possibly decarboxylated SAM (S-adenosylmethionamine)**,** to facilitate polyamine biosynthesis and its consequent antioxidant activity. Either way, this observation provides new insights into the interplay between oxidative stress response and polyamine biosynthesis in basidiomycetes and *A. mellea*, in particular. 

Valosin-containing proteins (VCPs) in higher eukaryotes are a key component of cellular processes involving protein ubiquitylation, both in proteasomal and in non-proteolytic pathways [49]. Am14558, a VCP, appeared to exhibit increased abundance under H_2_O_2_-induced oxidative stress (1.6-fold) (Table 1). This protein was also detected following menadione/FeCl_3_ treatment, exhibiting a minor decrease in abundance of 1.1-fold, indicating a distinct response to the two forms of oxidative stress. VCP (Am14558) has orthologues in yeast (Cdc48) and mammals (p97) and is a member of the AAA^+^ ATPase superfamily whose activities include proteolysis, DNA replication and membrane fusion [50]. VCP is also implicated in endoplasmic reticulum stress [51], ubiquitin regulatory processes, mitosis and DNA/RNA repair, interacting with a broad range of proteins [52]. ATPase activity of human VCP is inhibited by oxidative and nitrosative stress, via oxidation of cysteine 522 within the Walker A motif [53]. In yeast VCP (Cdc48), as in *A. mellea* (Am14558), this cysteine has been replaced with a threonine, rendering it resistant to inactivation by H_2_O_2_. In fact, mutation of the yeast threonine to cysteine actually increased the sensitivity of cells to H_2_O_2_, highlighting the role of this protein in protection against this form of oxidative stress [53].

Two peptidases were detected, with alternate changes in abundance in response H_2_O_2_. Am3212 (1.8-fold increased abundance; Table 1) is a cytosolic and mitochondrial zinc M3 metallopeptidase that acts on small peptides, containing 9–17 amino acids [54]. M3 peptidases are also known as mitochondrial intermediate peptidases (MIPs) and are involved in processing nuclear-encoded proteins targeted to the mitochondria. N-terminal presequence peptides cleaved from these proteins are further hydrolysed by MIPs [55]. These enzymes are highly conserved and have been shown to contribute to the pathogenicity of *Mycosphaerella graminicola*, a wheat pathogen [56]. One possibility is that increased expression of Am3212 is consequent to H_2_O_2_ presence, which mimics the host-defence environment that *A. mellea* experiences during plant infection. A second peptidase, Am16706, shows homology to insulin-degrading enzyme (IDE), an M16 peptidase involved in degrading insulin and amyloid-β in human cells. This enzyme is inhibited by H_2_O_2_, through cysteine oxidation, leading to oligomerisation and increased susceptibility to proteolysis [57,58]. Am16706 exhibited decreased abundance (2.3-fold) in *A. mellea* under H_2_O_2_-induced oxidative stress (Table 1), indicating that inactivation and subsequent degradation of Am16706 may have occurred. Interestingly, Am16706 also shows homology to a putative metallopeptidase Ste23p, which processes fungal a-factor mating pheromone [59,60]. Recently, this protein has been shown to work in concert with Cym1 in yeast, to clear N-terminal presequence peptides from the mitochondria [61], an activity shared by Am3212. Therefore, Am3212 peptidase expression may have been induced to counteract the reduction in levels of Am16706. Furthermore, a LuxS/M16 peptidase-like metal binding domain was identified in Am16706, which catalyses the cleavage of the thioester bond of S-ribosylhomocysteine (SRH; a product of SAM methylation of macromolecules) to form homocysteine [62,63]. We speculate that Am16706 levels are decreased following oxidative stress to avoid overproduction of homocysteine, which is toxic at high levels [64]. Homocysteine is metabolized to methionine by methionine synthase; however, inactivation of this enzyme by H_2_O_2_ would lower this capacity [36]. Thus, reduced abundance of Am16706 under H_2_O_2_ stress in *A. mellea* could conceivably be a protective measure, to regulate homocysteine levels consequent to S-ribosylhomocysteine degradation. Together, these results highlight the influence of oxidative stress on methionine synthesis in *A. mellea*, while also impacting on protein degradation processes. An increase in polyamine biosynthesis may further help alleviate the effects of H_2_O_2_ through ROS scavenging.

In addition to profiling the oxidative stress response, proteomic characterization also revealed a remodelling effect associated with the growth of *A. mellea* on different matrices. A total of 640 proteins showed higher abundance or were uniquely detected in liquid PDB cultures, while 341 proteins were enriched in agar cultures (Table S1). Proteins with putative secondary metabolism associations were represented among these proteins with altered abundance, demonstrating the influence of growth matrix on SM cluster expression (Table 2). Metabolomic analysis echoed these changes to the proteome, with distinct profiles evident in each growth condition (Figure 3, Figures S1 and S2). MS/MS analysis revealed fragment ions (*m*/*z* 246.8, 264.8) that were common to a number of different molecules in the agar culture extracts. This may indicate a core structure shared by these molecules, which may in turn undergo several modifications to yield molecules with distinct *m*/*z* and retention times. To the best of our knowledge, these molecules have not yet been identified in *A. mellea* metabolomic studies, and future investigations will aim to characterise these features. Previously, we have demonstrated that MS-based proteomics can be used as a prediction tool for SM production in *A. fumigatus* [65], whereby known metabolites were detected corresponding to detected cluster proteins. While studies are emerging on the activity and potential applications of SMs produced by *A. mellea* [66,67,68], relatively few have focused on the clusters and pathways involved in their production [28,30]. Bioinformatically-assigned putative SM clusters can be experimentally confirmed using gene deletions/mutations coupled with metabolomics [69]. Transcriptome analyses can also be utilized, to identify contiguous genes with similar expression patterns that are associated with an SM backbone enzyme [70,71]. In the present study, an analogous approach could be taken, utilising proteomic data to determine potential SM cluster members and define cluster borders. The biosynthetic gene cluster (BGC) members assigned by JGI were located within the data followed by genome walking to identify proteins with similar abundance profiles directly up/down stream. This approach revealed analogous protein abundance profiles in the case of three potential BGCs (Clusters 1.4, 1.11 and 1.29; Table S2). This represents a mechanism by which to define or confirm the borders of secondary metabolite clusters, which can be used as an alternative or adjunct to transcriptomic analyses.

Much of the secondary metabolite research conducted on *A. mellea* to date has focused on the melleolides, a family of molecules characterized by an orsellinic acid group coupled to a sesquiterpene by an ester linkage. These molecules are attractive research interest due to their associated bioactivity, which ranges from antifungal to cytotoxic [67,68]. Enzymes associated with melleolide biosynthesis have been identified, including protoilludene synthase (Pro1; Am14852) and the polyketide synthase ArmB (Am14842) [30,72]. ArmB has been shown to synthesise orsellinic acid (OA), while also exhibiting coupling activity, facilitating the ligation of OA to sesquiterpene alcohols, such as protoilludene derivatives [30]. While neither ArmB nor Pro1 were detected in the proteomic analyses, protein products of several neighbouring genes (Am14848, Am14849, Am14850, Am14855, Am14858, Am14860) were detected. Interestingly, all of these proteins exhibited higher abundance during growth in agar cultures, which could be indicative of co-regulation of the respective genes in this condition. Gene products flanking this region (Am14845 and Am14864) showed no significant changes in abundance. Similarly, Am14860 exhibited significantly higher abundance during agar growth, but this corresponded to a fold change of only 1.6, so inclusion of this hexokinase in the cluster could be more tentative. These results may help to putatively define the borders of Cluster 1.29, based on similar abundance patterns of the proteins detected. In line with this, two additional trends in abundance were found in relation to putative Clusters 1.4 and 1.11. The JGI-assigned Cluster 1.4 includes an acetyl-synthetase (Am14526) and a PKS (Am14527), detected in this study. In addition to these proteins, another PKS (Am14528) and an actin-like ATPase (Am14525) were detected, which all showed increased abundance in agar compared to liquid culture. Finally, five potential members from the putative Cluster 1.11 were identified, also with higher abundance in agar culture. These included dynein intermediate chain (Am18588), terpenoid cyclases/protein prenyltransferase (Am18591) and an aldo-ketoreductase (Am18600). To the best of our knowledge, no clusters have been experimentally defined in *A. mellea* to date, although some studies have characterized individual enzymes responsible for key steps in melleolide biosynthesis [28,30,72]. While the results presented herein do not definitively identify cluster boundaries in *A. mellea*, they do lay the foundation for future studies. Protoilludene synthesis has been shown in *A. gallica*, with a homolog of this enzyme associated with Cluster 1.29 [72]. Thus, while Cluster 1.29 has been linked to melleolide biosynthesis, products of the remaining putative BGCs have not yet been identified. These results present a target for future investigations to confirm the presence of these clusters, to identify their products and to investigate any associated bioactivity. 

We conclude that 2-DE and LC-MS/MS approaches can be applied to reveal new biology in a major plant pathogen for which minimal proteomic information is available. It is also clear that *A. mellea* can undergo rapid proteomic remodelling (within 3 h) in response to alternate oxidative stress conditions, resulting in significant changes in the abundance of enzymes involved in methionine and polyamine biosynthesis, as well as homocysteine production. Protein degradation pathways appear to be affected by H_2_O_2_-induced oxidative stress in *A. mellea*. The proteome of *A. mellea* also reveals alterations in abundance of putative secondary metabolism-related proteins when grown on agar compared to liquid culture. Changes to the metabolome under these differing growth conditions could be used as a tool to identify new metabolites from this phytopathogen and potentially identify SM clusters in *A. mellea*.

## Figures and Tables

**Table 1:** Details of unambiguously-identified *A. mellea* mycelial proteins found to be differentially abundant following H_2_O_2_ or menadione/FeCl_3_-induced oxidative stress for 3 h. Proteins from 2-DE identified by SameSpots™ software, analysed by LC-MS/MS with subsequent interrogation of the *A. mellea* cDNA database.

			H_2_O_2_		Menadione/FeCl_3_	
Spot No.	^a^ Accession No.	^b^ BLAST Description	ANOVA (*p*)	^c^ Fold Change	ANOVA (*p*)	^c^ Fold Change
339/398	Am17277	Cobalamin-independent methionine synthase	0.032	↑ 2.7	0.028	↑ 2.6
352	Am17277	Cobalamin-independent methionine synthase	0.005	↑ 1.8		
409	Am3212	Zinc metallopeptidase found in the cytoplasm and intermembrane space of mitochondria	0.008	↑ 1.8		
363	Am14050	Saccharopine dehydrogenase	0.036	↑ 1.7		
249	Am14558	Valosin-containing protein	0.017	↑ 1.6		
295	Am18454	Heat shock protein	0.032	↑ 1.6		
803	Am19877	Glutamic oxaloacetic transaminase aat1			0.011	↑ 1.5
178	Am16706	A-pheromone processing metallopeptidase ste23	0.044	↓ 2.3		
675	Am19873	Translation elongation factor 1a	0.012	↓ 1.8		
479	Am7452	Heat shock protein 90	0.034	↓ 1.5		

^a^ Accession number from the *A. mellea* cDNA database; ^b^ BLAST annotation following B2G analysis of proteins identified from the *A. mellea* cDNA database; ^c^ fold increase (↑) or decrease (↓) of protein following treatment.

**Table 2:** Differential abundance of putative secondary metabolism-related proteins identified in liquid and/or agar cultures of *A. mellea*.

^a^ Accession No.	^b^ BLAST Description	*p*-Value	^c^ Fold Change	Uniquely Detected	Unique Peptides	^d^ Cluster No.	Putative Product
Increased in Agar							
Am6587	Fatty acid synthase	0.002	5.8		111	1.1	
Am14527	Polyketide synthase	ns		Agar	3	1.4	
Am14528	Polyketide synthase	ns		Agar	2	1.4	
Am18600	Aldo keto reductase	ns		Agar	2	1.11	
Am19612	Acetyl-synthetase	ns		Agar	5	1.22	
Am15263	Acetyl-synthetase	ns		Agar	4	1.24	
Am315	Polyketide synthase	ns		Agar	7	1.25	
Am14855	Cytochrome P450	ns		Agar	2	1.29	Protoilludene/melleolides [28]
Increased in Liquid							
Am19046	RNA-binding domain-containing	0.016	2.7		11	1.3	
Am14843	NAD(P)-binding	0.015	2.2		10	1.28	Orsellinic acid/melleolides [29,30]
No significant Change							
Am19045	Glycosyltransferase family 20	ns			13	1.3	
Am14526	Acetyl-synthetase	ns			18	1.4	
Am12922	Alpha aminoadipate reductase Lys1	ns			12	1.9	
Am18601	Glycoside hydrolase family 7	ns			5	1.11	
Am10842	Acetyl-synthetase	ns			6	1.17	
Am20064	T-complex 1	ns			9	1.19	
Am20065	N-myristoyl transferase	ns			8	1.19	
Am14845	NAD P-binding	ns			17	1.28	Orsellinic acid/melleolides [29,30]

^a^ Accession number from the *A. mellea* cDNA database; ^b^ BLAST annotation following B2G analysis of proteins identified from the *A. mellea* cDNA database; ^c^ fold change of protein following treatment; ^d^ Putative secondary metabolite clusters retrieved from http://genome.jgi.doe.gov/Armme1_1/Armme1_1.home.html.

## Supplementary Materials

The following are available online at www.mdpi.com/2076-2607/5/3/60/s1, Table S1: Proteomic analyses of *A. mellea* grown in potato dextrose liquid and agar cultures. Table S2: Differential abundance of *A. mellea* proteins flanking putative secondary metabolism (SM) proteins. Figure S1: RP-HPLC profiles of metabolites from *A. mellea* grown in liquid and agar cultures. Figure S2: LC-MS/MS analysis of extracts from *A. mellea* liquid and agar cultures.
