# Supplementary material for: Proteomic Characterization of Armillaria mellea Reveals Oxidative Stress Response Mechanisms and Altered Secondary Metabolism Profiles
Source: Microorganisms. 2017 Sep 17;5(3):60. doi: 10.3390/microorganisms5030060 (PMC5620651; doi:10.3390/microorganisms5030060)
Supplement: Supplementary file 1 [file microorganisms-05-00060-s001.zip › supplementary/Supplementary Figures S1 and S2.docx]

Supplementary Figures

Proteomic characterization of *Armillaria mellea* reveals oxidative stress response mechanisms and altered secondary metabolism profiles.

Cassandra Collins, Rachel Hurley, Nada Almutlaqah, Grainne O’Keeffe, Thomas M. Keane, David A. Fitzpatrick and Rebecca A. Owens


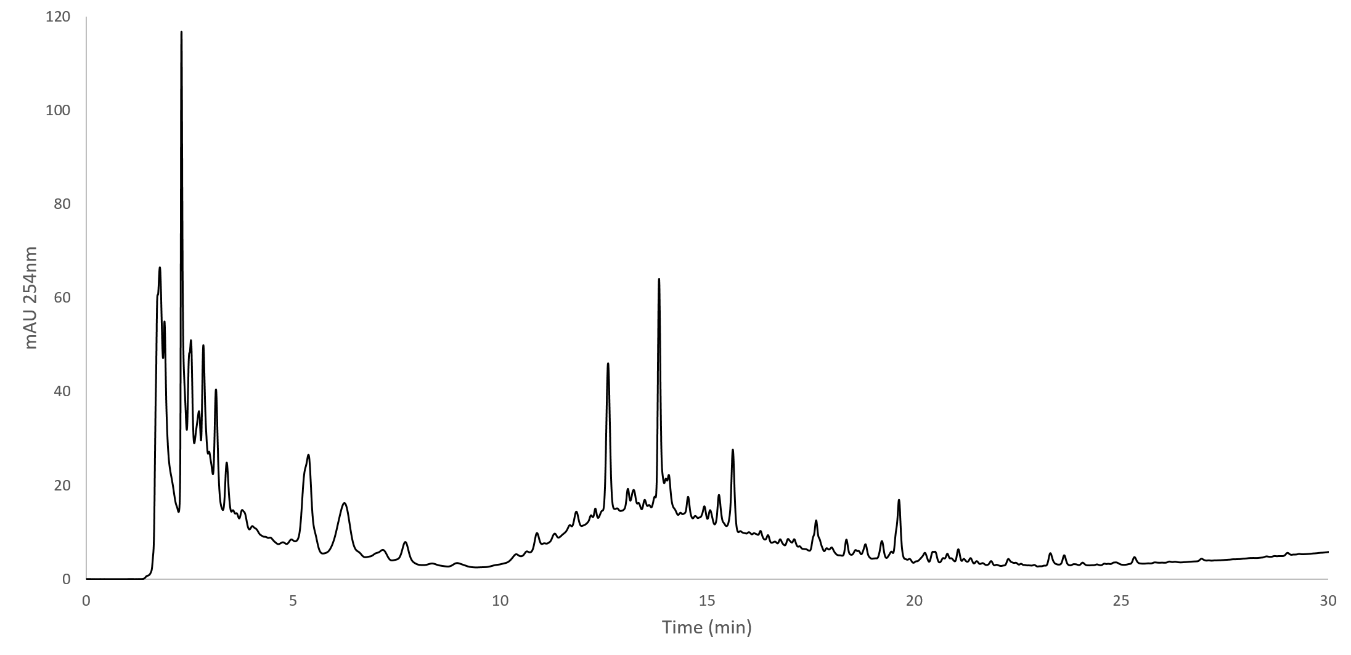


**(a)**


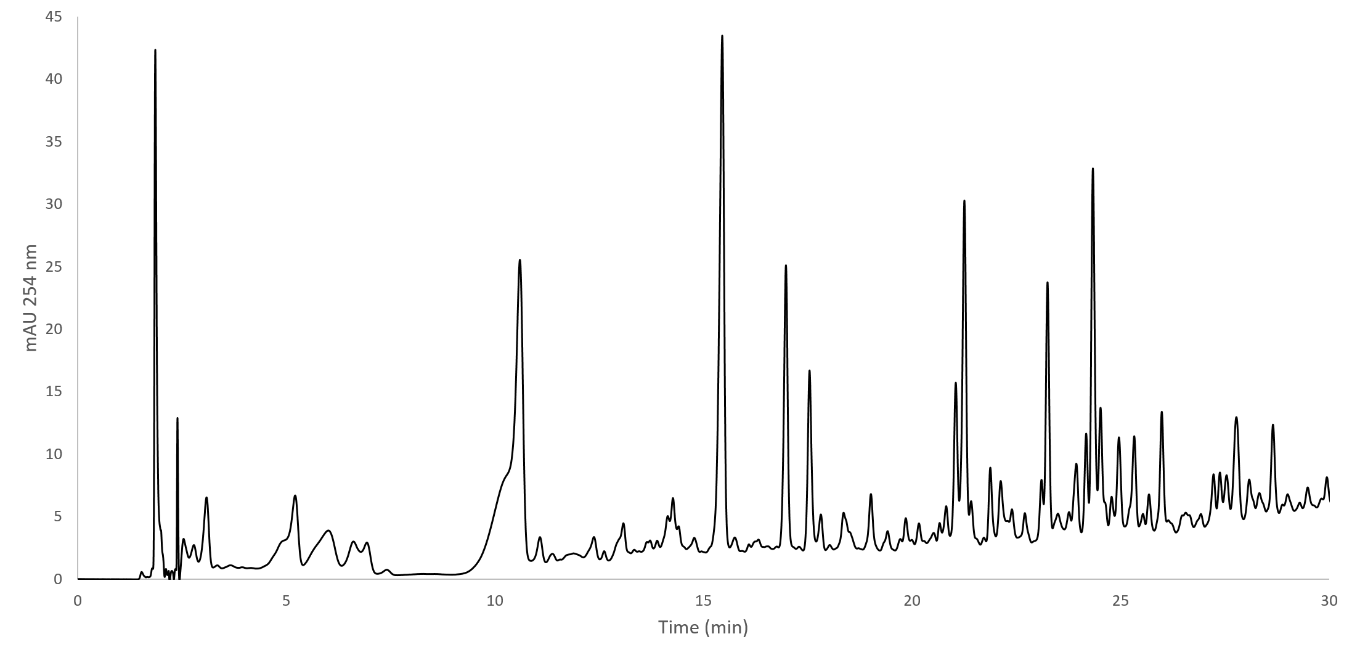


**(b)**

**Supplementary figure S1.** RP-HPLC profiles of metabolite extracts from (a) supernatant of liquid PDB cultures and (b) PDA solid state cultures of *A. mellea*. UV trace was monitored at 254 nm.

(a)


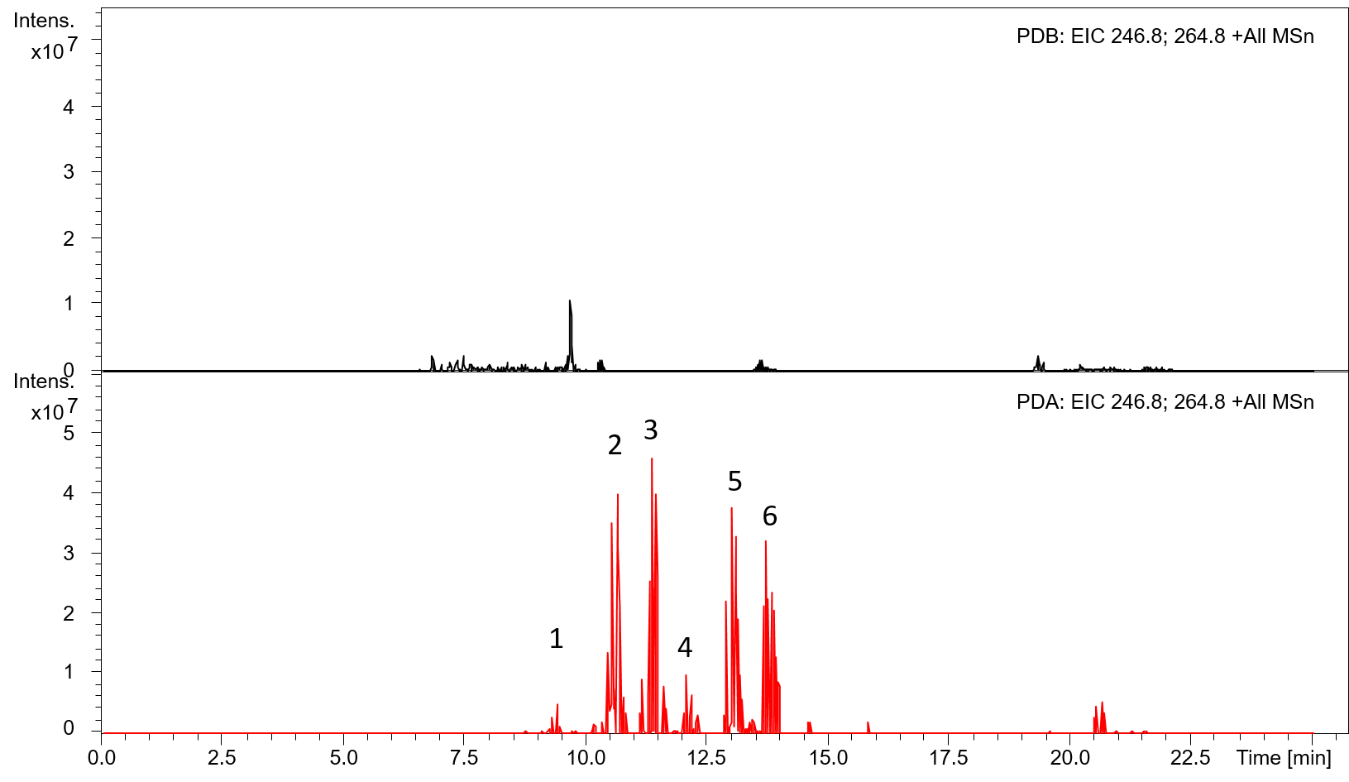


(b)


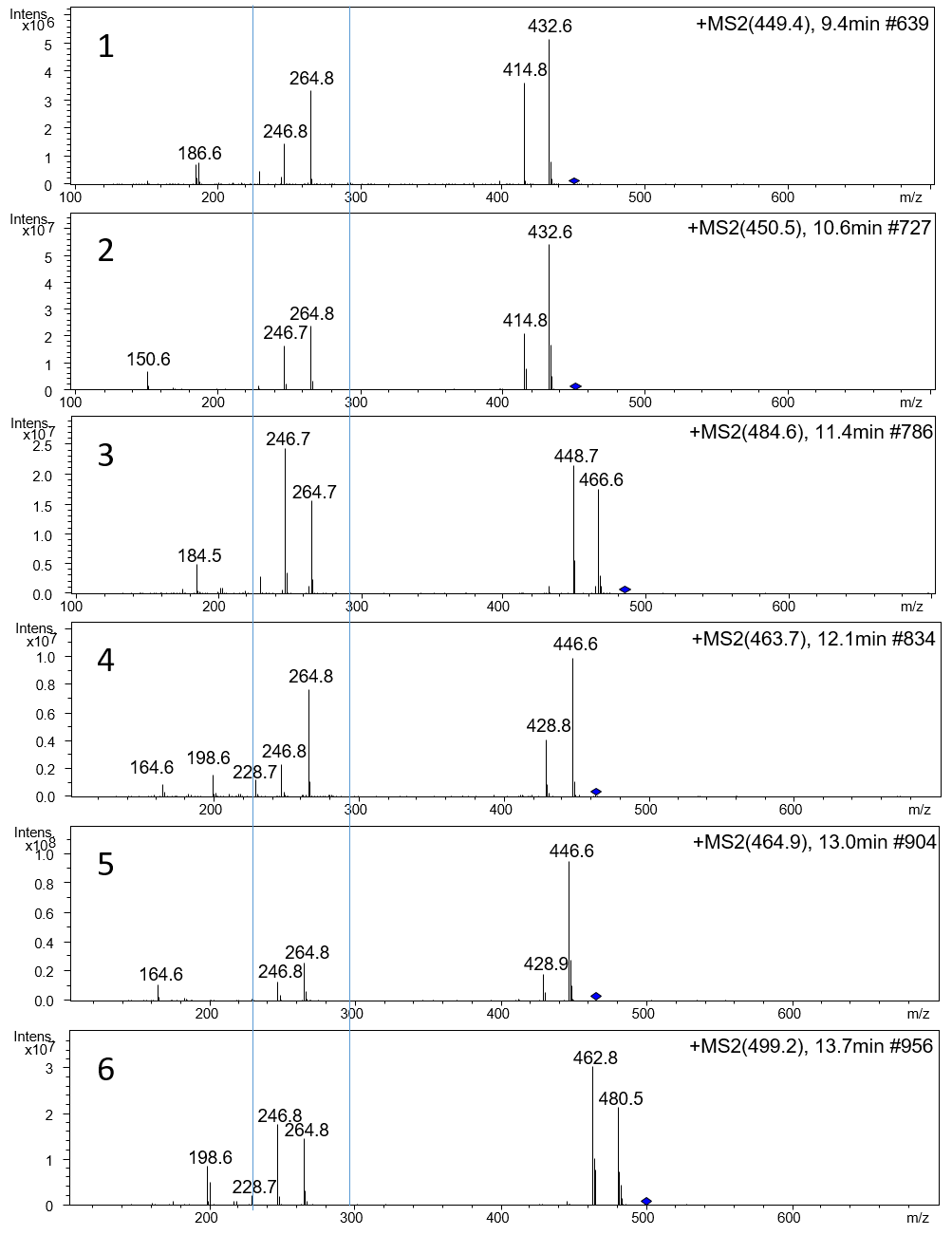


**Supplementary figure S2.** (a) Extracted ion chromatographs (EIC) profiling molecules with characteristic m/z 264, 248 fragments in MS/MS. Individual molecules containing these features are numbered **1**-**6**. (c) MS/MS spectra of molecules **1**-**6,** generating characteristic fragment ions (flanked by blue lines).
